# Supplementary figures and images for: Species comparison of liver proteomes reveals links to naked mole-rat longevity and human aging
Source: BMC Biol. 2018 Aug 2;16:82. doi: 10.1186/s12915-018-0547-y (PMC6090990; doi:10.1186/s12915-018-0547-y)

**A**

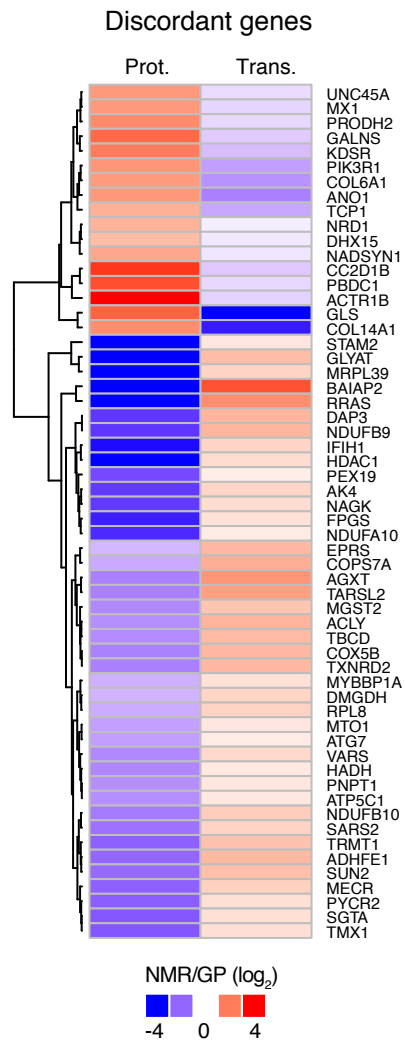

**B**

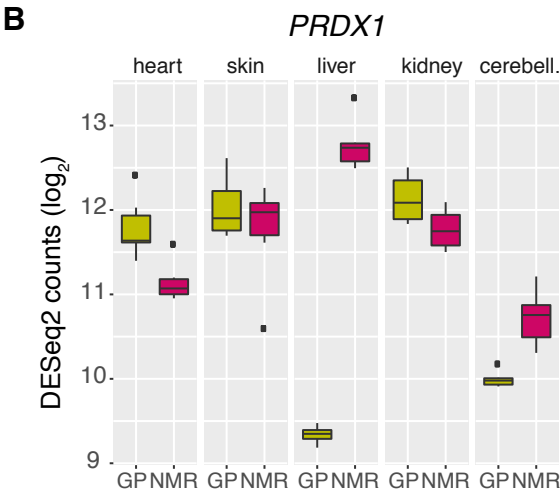

**C**

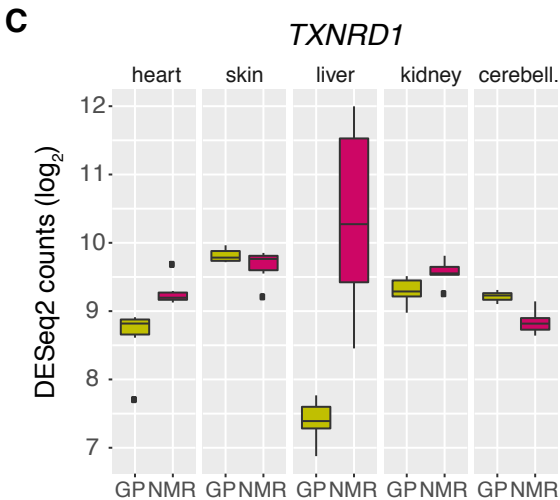

Supplement: Supplementary file 3 — Figure S1. (A) Heatmap showing genes that displayed discordant NMR vs. GP fold changes at the transcript and protein level. Only cases significant in both comparisons (q < 0.1) are shown. (B and C) Comparison of transcript levels of PRDX1 and TXNRD1 across multiple tissues. For both genes, transcript levels are increased in NMR vs. GP in the liver (q < 2.2 × 10− 300 for PRDX1; q = 1.5 × 10− 45 for TXNRD1), while they show similar abundances in the other tissues examined. RNAseq data for heart, skin, kidney and cerebellum were obtained from [34]. Related to Fig. 1 and Additional file 1: Table S1 and Additional file 2: Table S2. (PDF 488 kb) [file 12915_2018_547_MOESM3_ESM.pdf]

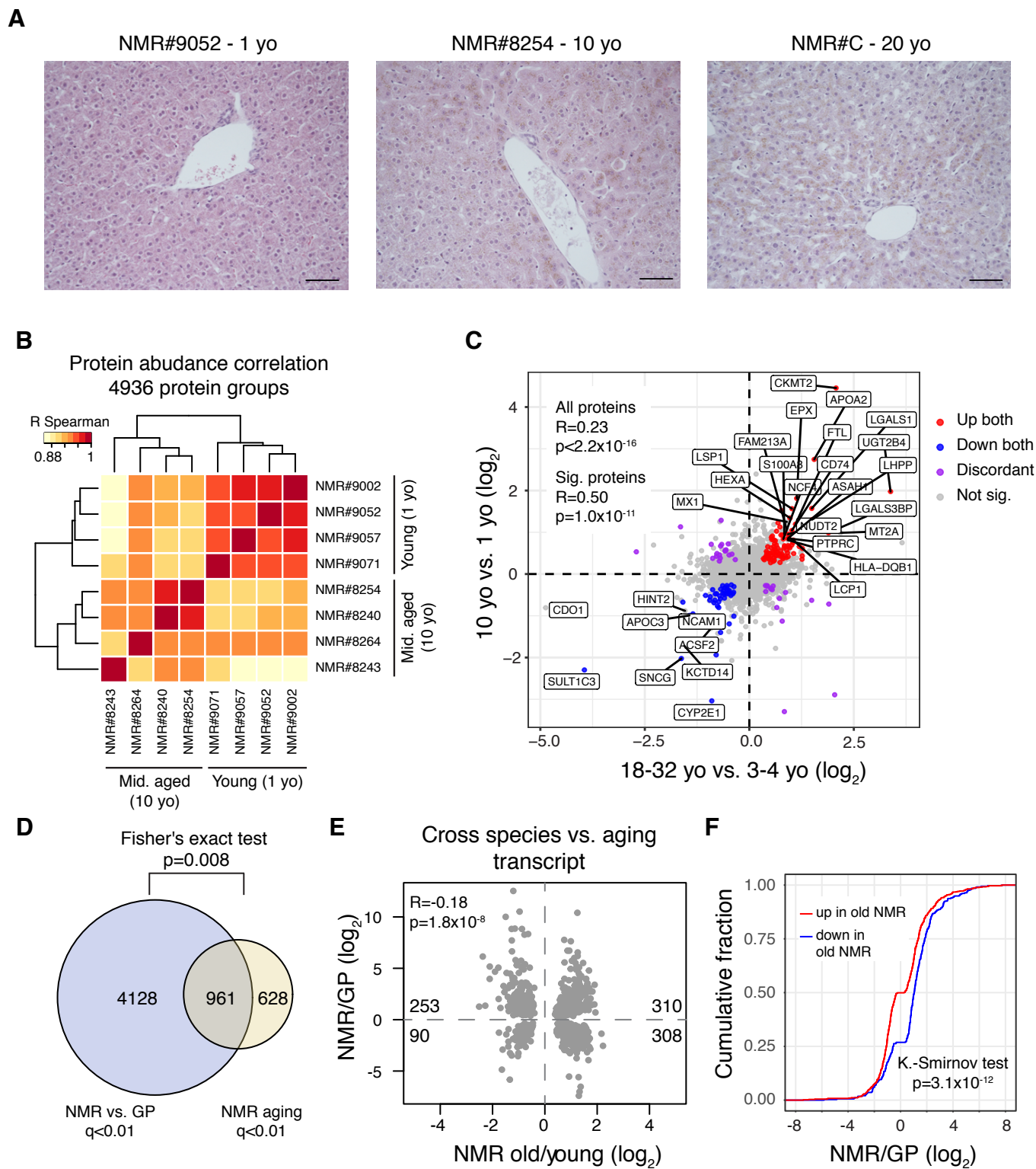

Supplement: Supplementary file 6 — Figure S2. Comparison of liver proteomes between middle-aged and young NMRs, and correlation analysis of transcript level differences in NMR vs. GP and NMR aging. (A) Representative micrographs show H&E stained (FFPE) liver tissue sections from NMRs of the indicated age groups. Scale bar = 100 μm. (B) Livers from 4 young (1 yo) and 4 middle-aged (> 10 yo) NMRs were compared by Tandem Mass Tags (TMT) based quantitative mass spectrometry. Hierarchical clustering based on the correlation between proteome profiles based on 4936 protein groups cross-quantified between the two age groups (Additional file 5: Table S4). (C) Comparison of protein fold changes calculated between old vs. young (x-axis) and middle-aged vs. young (y-axis) NMRs. Colored dots indicate proteins significant (q < 0.1) in both comparisons. The names of selected proteins that show consisted fold changes in the two comparisons are indicated. (D) Significant overlap between differentially expressed genes (DEGs) in NMR vs. GP and in aging of NMR. (E) Comparison between cross-species and aging-related fold changes for the 875 DEGs significant in both comparisons (q < 0.01) shows significant negative correlation. (F) Cumulative distributions of NMR vs. GP fold changes for the 875 DEGs also significantly up- (red) or down- (blue) regulated in NMR aging. The x-axis was restricted to ±8 for display purpose. Related to Fig. 3, Additional file 5: Table S4 and Additional file 8: Table S6. (PDF 13567 kb) [file 12915_2018_547_MOESM6_ESM.pdf]

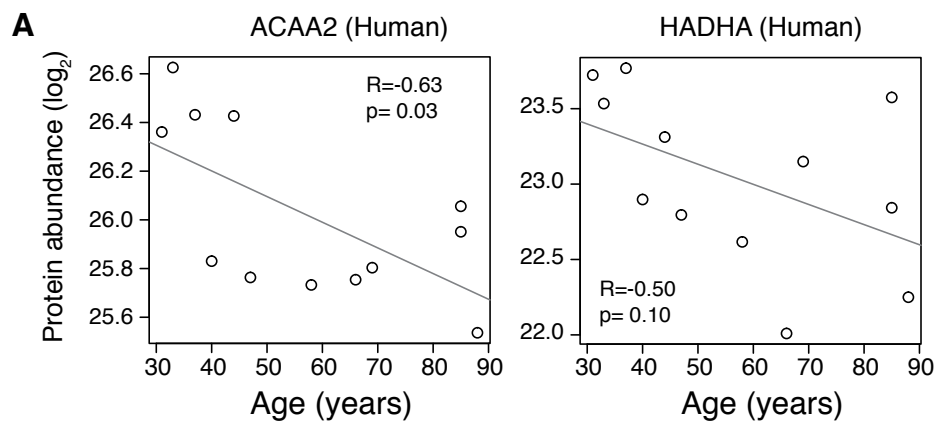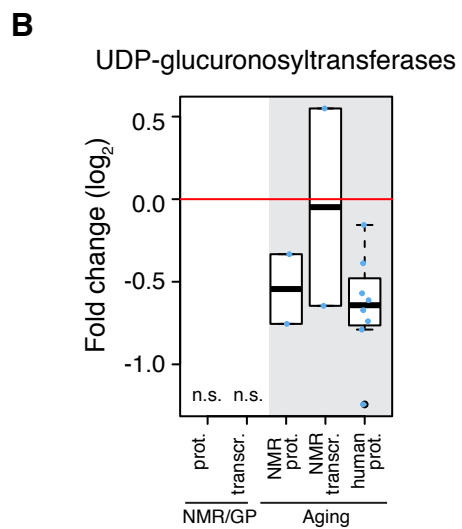

Supplement: Supplementary file 10 — Figure S3. Examples of enzymes involved in fatty acid beta-oxidation and xenobiotic metabolism that decrease during aging in human liver. (A) Additional examples of enzymes involved in lipid metabolism decreasing during aging in human liver. (B) Detoxifying enzymes decreasing during aging in both NMR and humans. Only significantly affected genes are shown; cut offs: NMR aging, combined q < 0.05; human proteome aging q < 0.1; n.s. = no significant cases detected. Related to Fig. 4. (PDF 386 kb) [file 12915_2018_547_MOESM10_ESM.pdf]

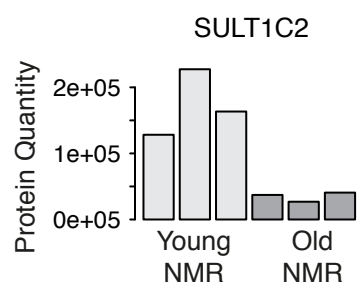

Supplement: Supplementary file 11 — Figure S4. SULT1C2 decreases with aging in NMR. Related to Fig. 5. (PDF 328 kb) [file 12915_2018_547_MOESM11_ESM.pdf]
